# The impact of high-intensity interval training on vascular function in adults: A systematic review and meta-analysis

**DOI:** 10.3389/fcvm.2022.1046560

**Published:** 2022-11-17

**Authors:** Mousa Khalafi, Mohammad Hossein Sakhaei, Fatemeh Kazeminasab, Michael E. Symonds, Sara K. Rosenkranz

**Affiliations:** ^1^Department of Physical Education and Sport Sciences, Faculty of Humanities, University of Kashan, Kashan, Iran; ^2^Department of Exercise Physiology, Faculty of Sport Sciences, University of Guilan, Guilan, Iran; ^3^Academic Unit of Population and Lifespan Sciences, Centre for Perinatal Research, School of Medicine, University of Nottingham, Nottingham, United Kingdom; ^4^Department of Kinesiology and Nutrition Sciences, University of Nevada, Las Vegas, Las Vegas, NV, United States

**Keywords:** high-intensity interval training, moderate-intensity continuous training, vascular function, cardiovascular diseases, metabolic disorders

## Abstract

**Aim:**

We performed a systematic review and meta-analysis to investigate the effects of high-intensity interval training (HIIT) compared with moderate-intensity continuous training (MICT) or with no exercise (CON) on vascular function in adults who were free of cardiometabolic diseases and those with cardiometabolic diseases.

**Methods:**

A search across three electronic databases including Scopus, PubMed, and Web of Science was conducted through February 2022 to identify the randomized trials evaluating HIIT vs. MICT and/or CON on vascular function as measured using brachial artery flow-mediated dilation (FMD) in adults. Separate analyses were conducted for HIIT vs. MICT and/or CON to calculate weighted mean differences (WMD) and 95% confidence intervals (95% CIs) using random or fixed models.

**Results:**

A total of 36 studies involving 1,437 participants who were either free of cardiometabolic diseases or had cardiometabolic diseases were included in the meta-analysis. HIIT effectively increased FMD when compared with MICT [1.59% (95% CI 0.87–2.31), *p* = 0.001] or CON [3.80% (95% CI 2.58–5.01), *p* = 0.001]. Subgroup analysis showed that HIIT increased FMD in participants with cardiovascular and metabolic diseases, but not in participants who were free of cardiometabolic diseases. In addition, HIIT effectively increased FMD regardless of age and body mass index.

**Conclusion:**

We confirm that HIIT is effective for improving vascular function in individuals with metabolic disorders and cardiovascular diseases and has a superior effect compared to MICT, demonstrating time efficiency.

**Systematic review registration:**

[https://www.crd.york.ac.uk/prospero], identifier [CRD42022320863].

## Introduction

Vascular function, determined by brachial artery flow-mediated dilation (FMD), plays a key role in circulating cardiovascular homeostasis, which is critical for cardiovascular health (1, 2). Dysfunction of vascular endothelium determines the pathogenesis of atherosclerosis and contributes to the development of clinical cardiovascular diseases (3–5). Endothelial dysfunction is characterized by an imbalance between vasodilation and vasoconstriction and promoting inflammation, oxidative stress, and reduced production of nitric oxide (NO) (2). FMD is the gold standard technique to assess vascular function and is widely used to measure endothelial health (6), and is an independent predictor of cardiovascular disease (7, 8). It is an important therapeutic and preventive target in the management of cardiovascular disease.

There is growing evidence showing the beneficial effects of exercise training in mitigating some adverse effects of cardiovascular disease, with both aerobic and strength training (9–11). These include the modulation of inflammatory markers (12–14), lipid profiles (15), visceral fat mass (16, 17), markers of glycemia (18, 19), and vascular function (20, 21) in both healthy populations and those with chronic disease. Moderate-intensity continuous training (MICT) is often recommended to improve cardiometabolic health, with current physical activity guidelines recommending a minimum 150 min of moderate-intensity (∼40–60% VO_2__max_) or 75 min of vigorous-intensity (∼60–85% VO_2__max_) physical activity per week (22–24) or longer to prevent excess weight gain or reduce body weight (25). Despite the beneficial effects of continuous training, lack of time, poor adherence, and low motivation can limit engagement. Higher intensity exercise is associated with greater benefits, although it can be difficult to maintain for some people. Enjoyment of exercise training and adherence are important for effectiveness in health care-based interventions (26, 27). As such, high-intensity interval training (HIIT) characterized by alternating short bouts of high-intensity exercise with active or passive recovery periods, has been proposed as an alternative time-efficient method of training (28, 29). Emerging evidence from systematic reviews and meta-analyses demonstrate beneficial effects of HIIT on cardiometabolic risk factors (30). As such, HIIT improves body composition (31), visceral (17), and liver (32) fat mass, some inflammatory markers (13), glycemic markers (33–35), and several chronic pain conditions (36). In addition, HIIT may be an effective approach to improve vascular function. In this regard, a 2015 meta-analysis that included seven randomized trials with 182 patients with cardiovascular and metabolic diseases, indicated a significant increase in FMD compared with MICT (37). However, the effect of HIIT on vascular function compared with non-exercise controls (CON) was not investigated. Many new studies have been published that enable the influence of health status (free of cardiometabolic diseases vs. with cardiometabolic diseases), body mass index (BMI) and age, as well as type, duration, and volume of HIIT to be examined. Therefore, the current systematic review and meta-analysis investigated the effect of HIIT on FMD as compared with either MICT or CON. In addition, we compared subgroup analyses to investigate whether the health status (free of cardiometabolic diseases, metabolic disorders, and cardiovascular diseases), BMI, age, interval types, intervention duration, and volume of intense bouts of HIIT influenced the FMD.

## Methods

### Trial registration

The current meta-analysis was conducted according to the 27-item PRISMA guidelines (Preferred Reporting Items for Systematic Reviews and Meta Analyses) and the Cochrane Handbook of Systematic Reviews of Interventions. The systematic review and meta-analysis was registered prospectively (ID: CRD42022320863).

### Search strategy

The search was conducted across three electronic databases including Scopus, PubMed, and Web of Science. Two independent reviewers (MK and MHS) identified published articles until February 2022. Records were searched using two sets of keywords. The operator “AND” was used to link terms, and synonyms were joined with the operator “R.” The search strategy for identifying studies with HIIT included “high intensity interval training,” “high intensity interval exercise,” “high intensity intermittent exercise,” “aerobic interval training,” “aerobic interval exercise,” “interval training,” “sprint interval training,” and “sprint interval exercise.” In addition, the search strategy for identification of studies with FMD included “brachial artery,” “brachial artery dilation,” “flow mediated dilation,” “endothelial function,” “endothelium,” “artery blood flow,” “artery dilation,” “flow-mediated,” “flow mediated,” “vascular,” “vascular endothelium,” “vascular reactivity,” and “vasodilation.” There were no limitations on publication dates, but the search was limited to English language articles, human research, and article type. Search term combinations used in each database are presented in Supplementary Table 1.

### Eligibility criteria and study selection

**FIGURE 1:**
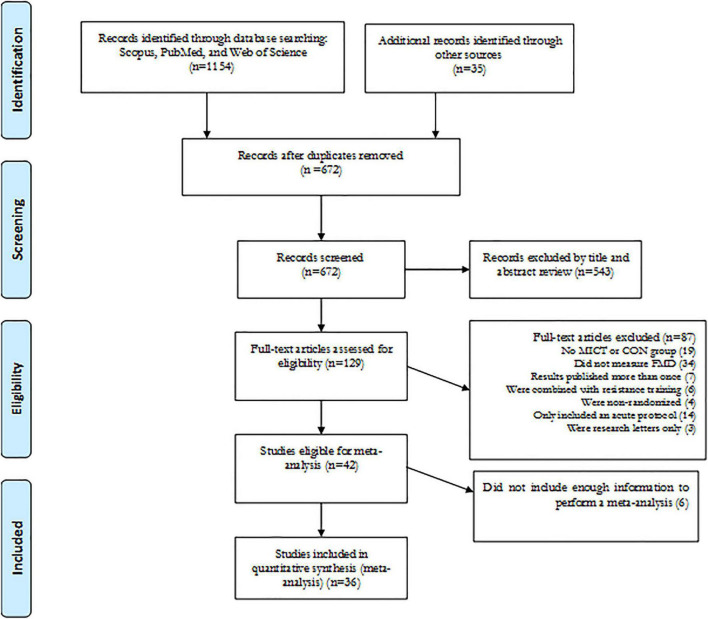
Flow diagram of systematic literature search and article selection.

Following removal of duplicate publications, studies were screened by title and abstract and then the full texts of potentially eligible studies were reviewed by two independent reviewers (MK and MHS) and any disagreements were resolved by discussion with another author (FK). Studies were included if they met the following criteria: (1) English language articles, (2) peer-reviewed, full-text articles, (3) studies with human participants, ages ≥18 years, (4) studies that included HIIT and MICT and/or control with randomized designs, (5) studies with intervention durations of at least 2 weeks, and (6) studies that included assessments of FMD at pre- and post-intervention or change scores. HIIT refers to repeated high-intensity exercise intervals performed at 80–100% of HR_peak_, interspersed with recovery periods of complete rest or light exercise (38). Sprint interval training (SIT) was included as HIIT if exercise was characterized by “all-out” or “supramaximal” efforts (≥100% of maximal work rate or VO_2__max_) that was interspersed with recovery periods (39). For the HIIT intervention category, HIIT and/or SIT were included. Therefore, HIIT and SIT for subgroup analysis was categorized separately as longer-interval HIIT (LI-HIIE) and SIT. For MICT, aerobic-based exercise, including low-to-moderate intensity intervals or continuous training, were included. For exercise mode, running, walking, cycling, and elliptical exercise were included. For vascular function, studies were included where vascular function was measured using FMD with units of measurement expressed as percentages (%). Exclusion criteria included non-English language publications, non-original research, research letters, conference proceedings, case reports, short reports, and reviews. The flow diagram of the systematic search process is presented in Figure 1.

### Data extraction and synthesis

Data extraction was conducted by two independent authors (FK and MHS) and any disagreements were resolved through discussion with another author (MK). Data extracted from each study included the following: (a) study characteristics including first author names, year of publication, study design, and sample size, (b) participant characteristics including age, sex, BMI, and health status, (c) intervention characteristics including exercise mode and classification (HIIT, SIT, or MICT), duration, frequency, and total time of volume of exercise a week, and (d) vascular function outcome assessment methodology. For each outcome of interest, pre- and post-intervention values (means and SDs) or mean differences and associated SDs were extracted. When required, means and SDs were calculated from standard errors, 95% confidence intervals (95% CIs), medians, ranges, or interquartile ranges (40–42). In addition, when required, data extraction from figures was performed using Getdata Graph Digitizer software. In addition, to compare the total minutes of HIIT vs. MICT per week (min), weekly total exercise time was calculated, including the warm-up and cool-down time. Also, volume of intense bouts of HIIT per session (bout duration × repetitions) and week (bout duration × repetitions × sessions per week) were calculated and categorized as HIIT volume (weekly total time of ≤30 min vs. >30 min).

### Quality assessment and sensitivity analyses

The methodological quality of included studies was assessed using the Physiotherapy Evidence Database (PEDro) tool. We excluded 2 items (for non-blinding of participants and intervention) from the original 11-item scale, because participants and intervention providers could not be blind to the assigned exercise conditions during studies. Therefore, study quality was assessed based on 9 items (eligibility criteria, random allocation of participants, assessed outcomes in 85% of participants, baseline comparison, allocation concealment, intention-to-treat analysis, reporting of statistical comparisons between groups, and point estimates and variability statistics). PEDro scores were determined by one reviewer (MK) and verified by another (FK) (Supplementary Table 2). Sensitivity analyses were performed by omitting each study individually to determine whether results changed.

### Statistical analyses

Meta-analyses were conducted using version 2.0 of the Comprehensive Meta-analysis (CMA) software (Biostat Inc., NJ, USA). Two separate analyses were performed for comparing the effects of (1) HIIT vs. MICT, and (2) HIIT vs. CON on FMD. Analyses were conducted through weighted mean differences (WMD) and 95% CIs using fixed or random effects models. Several subgroup analyses were conducted as follows: health status (free of cardiometabolic diseases, metabolic disorders, and cardiovascular diseases), mean BMI (BMI <30 kg/m^2^ vs. ≥30 kg/m^2^), mean age (age <0 years vs. ≥50 years), interval types (HIIT vs. SIT), intervention duration (intervention <12 weeks-short-term vs. ≥12 weeks-medium-term), and volume of intense bouts of HIIT (weekly total time of ≤30 min vs. >30 min). In addition, a subgroup analysis was performed based on type of HIIT (LI-HIIT and SIT), matching of work performed for HIIE vs. MICT (matched work vs. un-matched work). Significance was set at *p* < 0.05. The *I*^2^ statistic was used to determine the heterogeneity, and *I*^2^ values were defined as follows: 25, 50, and 75% indicated low, moderate, and high heterogeneity, respectively. Based on *I*^2^ values, fixed models were used when *I*^2^ values were lower than 25%, and random effects models were used when *I*^2^ values were higher than 25%. Finally, publication bias was assessed using visual interpretation of funnel plots with Egger’s tests performed as secondary assessments were significant publication bias was considered at *p* < 0.1 (43).

## Results

### Included studies

Our initial search strategy revealed 454 records from Scopus, 314 records from PubMed, and 386 records from Web of Science. After eliminating duplicates and screening titles and abstracts, 129 articles were included in the full-text analysis based on inclusion and exclusion criteria. After reviewing the full-texts, 93 studies were excluded according to the reasons presented in Figure 1. Finally, 36 studies met all eligibility criteria and were included in the meta-analysis, of which 18 studies compared HIIT vs. MICT (44–61), 10 studies compared HIIT vs. CON (62–71), and 8 studies compared HIIT vs. CON and MICT (72–79). In addition, only one included study used a crossover design (57). A majority of studies used cycling and others used running, walking, jogging and elliptical training.

### Participant characteristics

A total of 1437 adults were included with the range of sample sizes being 11 (57) to 200 (47). The mean age of participants ranged from 21 (65) to 75 years (79) and the mean BMI of participants ranged from 21 (65) to 37 kg/m^2^ (66). Both males and females were included in the majority of studies (44–48, 50–53, 55, 57–61, 64, 65, 68–77, 79), females only in five studies (49, 62, 63, 66, 67), and males only in two studies (54, 78). In the meta-analysis, participant health status varied regarding health and disease status, and included overweight and obesity, metabolic syndrome, prediabetes, type 2 diabetes, type 1 diabetes, polycystic ovarian syndrome, hypertension, heart failure, coronary artery disease, myocardial infarction, repaired tetralogy of Fallot (open heart surgery), and heart transplant. In addition, in subgroup analyses, obesity, type 2 diabetes, polycystic ovarian syndrome, type 1 diabetes, prediabetes, and the metabolic syndrome were included together as metabolic disorders (45, 48, 50, 54, 58, 59, 62, 63, 66, 67, 69, 70, 72–74, 77), and coronary artery disease, myocardial infarction, repaired tetralogy of Fallot, hypertension, heart failure, and heart transplant were included as cardiovascular diseases (44, 46, 47, 51, 52, 57, 60, 61, 68, 71, 75, 76, 79). If participants did not have any chronic disorders, they were included as free of cardiometabolic diseases (49, 53, 55, 56, 64, 65, 78) and full details of participant characteristics are shown in Table 1.

**TABLE 1:** Characteristics of the participants and interventions.

References	Sample size (sex)	Intervention	Participants characteristics	Age (years)	BMI (kg/m^2^)	Exercise program duration, type, and frequency	Intervention protocol (HIIT or SIT vs. MICT and CON)	Total minutes of high-intensity bouts per session and week	Total minutes of HIIT vs. MICT per week (min)
Abdi et al. (62)	30 (female)	HIIT CON	Type 2 diabetes	HIIT: 20−44 CON: 20−44	HIIT: 29.2 ± 1.3 CON: 28.7 ± 1.5	12 weeks treadmill running; unsupervised 3 days/week	HIIT: four sets of 4-min at 85–95% of HR_max_ with 3-min recovery at 50–60% of HR_max_ CON: maintained the activities of daily living without any training program	16 (48)	NA
Almenning et al. (63)	20 (female)	HIIT CON	Polycystic ovary syndrome	HIIT: 27.2 ± 5.5 CON: 27.2 ± 5.5	HIIT: 26.1 ± 6.5 CON: 26.5 ± 5.0	10 weeks treadmill or outdoor walking/running and/or cycling; supervised 1 day/week; unsupervised 2 day/week	HIIT: four sets of 4-min at 90–95% of HR_max_ with 3-min recovery at 70% of HR_max_ (2 sessions) + SIT: ten sets of 1-min at maximal intensity by 1-min recovery (1 session) CON: without any structure program	14 (42)	NA
Angadi et al. (44)	19 (male, female)	HIIT MICT	Heart failure	HIIT: 69.0 ± 6.1 MICT: 71.5 ± 11.7	HIIT: 29.8 ± 5.1 MICT: 29.3 ± 2.8	4 weeks treadmill training; unsupervised 3 days/week	HIIT: four sets of 2–4 min at 80–90% of HR_peak_ with 2–3 min recovery at 50% of HR_peak_ MICT (not matched-work with HIIT): 15–30 min at 60–70% of HR_peak_	16 (48)	48–85 vs. 45–90
Baekkerud et al. (45)	30 (male, female)	HIIT_1_ HIIT_2_ MICT	Obese and overweight	HIIT_1_: 45.0 ± 8.0 HIIT_2_: 39.0 ± 10.0 MICT: 41.0 ± 10.0	HIIT_1_: 30.8 HIIT_2_: 31.4 ± 5.3 MICT: 29.0 ± 2.7	6 weeks treadmill walking/running; supervised 3 days/week	HIIT_1_: ten sets of 1-min at 90% of HR_max_ with walking recovery HIIT_2_: four sets of 4-min at 85–95% of HR_max_ with 3-min recovery at 70% of HR_max_ MICT (matched-work with HIIT): 45-min at 70% of HR_max_	HIIT_1_: 10 (30) HIIT_2_: 16 (48)	57 and 75 vs. 135
Boff et al. (72)	36 (male, female)	HIIT MICT CON	Type 1 diabetes	HIIT: 26.1 ± 7.8 MICT: 23.7 ± 5.8 CON: 20.8 ± 2.6	HIIT: 23.2 ± 2.4 MICT: 24.1 ± 2.0 CON: 22.7 ± 2.6	8 weeks cycling; supervised 3 days/week	HIIT: 20-min at 50% of HR_max_ for first 2 weeks three to six sets of 1-min at 80–85% of HR_max_ with 4–5 min recovery at 50% of HR_max_ MICT (ND matched-work with HIIT): 20–40 min at 50 65% of HR_max_ CON: walking at least three times a week for 30 min	3–6 (9–18)	18–30 vs. 20–40
Bouaziz et al. (64)	60 (male, female)	HIIT CON	Sedentary older adults	HIIT: 72.9 ± 2.5 CON: 74.3 ± 3.4	HIIT: 28.7 ± 5.6 MICT: 28.8 ± 5.1	9.5 weeks cycling; supervised 2 days/week	HIIT: six sets of 4-min at first ventilator threshold with 1-min recovery at 40% of first ventilator threshold CON: sedentary lifestyle and current food habits	24 (48)	NA
Chidnok et al. (65)	24 (male, female)	HIIT CON	Healthy young adults	HIIT: 21.3 ± 0.7 CON: 21.3 ± 0.7	HIIT: 21.3 ± 3.9 CON: 21.3 ± 4.4	6 weeks cycling; supervised 3 days/week	HIIT: five sets of 1-min at 80% of HR_max_ with 2-min recovery CON: Maintained daily activities	5 (15)	NA
Currie et al. (46)	22 (male, female)	HIIT MICT	Coronary artery disease	HIIT: 62.0 ± 11.0 MICT: 68.0 ± 8.0	HIIT: 27.9 ± 4.9 MICT: 27.3 ± 4.2	12 weeks cycling; supervised 2 days/week	HIIT: ten sets of 1-min at 89% of peak power output with 1-min recovery at 10% of peak power output MICT (unmatched-work with HIIT): 30–50 min at 58% of peak power output	10 (20)	70 vs. 90–130
Conraads et al. (47)	200 (male, female)	HIIT MICT	Coronary artery disease	HIIT: 57.0 ± 8.8 MICT: 59.9 ± 9.2	HIIT: 28.0 ± 4.4 MICT: 28.5 ± 4.3	12 weeks cycling; supervised 3 days/week	HIIT: four sets of 4-min at 90–95% of HR_peak_ with 3-min recovery at 50–70% of HR_peak_ MICT (matched-work with HIIT): 47-min at 65–75% of HR_peak_	16 (48)	114 vs. 141
Ghardashi Afousi et al. (73)	75 (male, female)	HIIT MICT CON	Type 2 diabetes	HIIT: 54.8 ± 6.2 MICT: 53.1 ± 4.8 CON: 54.2 ± 5.6	HIIT: 29.4 ± 0.9 MICT: 28.9 ± 1.0 CON: 29.3 ± 1.3	12 weeks cycling; supervised 3 days/week	HIIT: twelve sets of 1.5-min at 85–90% of HR_max_ with 2-min recovery at 55–60% of HR_max_ MICT (matched-work with HIIT): 42-min at 70% of HR_max_ CON: Maintained daily activities	18 (54)	186 vs. 186
Gilbertson et al. (66)	25 (female)	HIIT CON	Obese	HIIT: 48.5 ± 13.7 CON: 45.7 ± 12.1	HIIT: 37.3 ± 7.2 CON: 37.8 ± 5.5	2 weeks cycling; supervised 6 days/week	HIIT: ten sets of 3-min at 90% of HR_*max/peak*_ with 3-min recovery at 50% of HR_*max/peak*_ CON: received nutrition advice	30 (180)	NA
Jo et al. (48)	37 (male, female)	HIIT MICT	Hypertensive, metabolic syndrome	HIIT: 49.9 ± 7.3 MICT: 51.8 ± 8.5	HIIT: 24.9 ± 2.8 MICT: 24.9 ± 3.2	8 weeks treadmill running; supervised 3 days/week	HIIT: 5-min at 60% of heart rate reserve followed by three sets of 3-min at 80% of heart rate reverse with 3-min at 40% of heart rate reserve MICT (matched-work with HIIT): 35-min at 60% of heart rate reserve	9 (27)	75 vs. 120
Klonizakis et al. (49)	22 (female)	HIIT MICT	Postmenopausal	HIIT: 64.0 ± 7.0 MICT: 64.0 ± 4.0	ND	2 weeks cycling; supervised 3 days/week	SIT: 10 sets of 1-min at 100% of peak power output with 1-min recovery at 30 W MICT (unmatched-work with HIIT): 40-min at 65% of peak power output	10 (30)	78 vs. 138
Lee et al. (67)	30 (female)	HIIT CON	Breast cancer	HIIT: 49.1 ± 7.9 CON: 44.7 ± 11.2	HIIT: 33.1 ± 7.6 CON: 30.1 ± 7.7	8 weeks cycling; supervised 3 days/week	HIIT: seven sets of 1-min at 90% of peak power output with 2-min recovery at 30% of peak power output CON: less than 30 min of total structured exercise per week	7 (21)	NA
Malin et al. (50)	26 (male, female)	HIIT MICT	Prediabetes	HIIT: 59.9 ± 7.6 MICT: 60.4 ± 8.6	HIIT: 30.9 ± 3.8 MICT: 35.6 ± 6.0	2 weeks cycling; unsupervised 6 days/week	HIIT: 10 sets of 3-min at 90% of HR_peak_ with 3-min recovery at 50% of HR_peak_ MICT (matched-work with HIIT): 60-min at 70% of HR_peak_	30 (180)	360 vs. 360
Mitranun et al. (74)	45 (male, female)	HIIT MICT CON	Type 2 diabetes	HIIT: 61.2 ± 10.5 MICT: 61.7 ± 10.1 CON: 60.9 ± 9.3	HIIT: 29.6 ± 1.9 MICT: 29.4 ± 2.6 CON: 29.7 ± 1.5	12 weeks treadmill running; supervised 3 days/week	HIIT: 20-min at 50% of VO_2__peak_ for 2 weeks four to six sets of 1-min at 80–85% of VO_2__peak_ with 4-min recovery at 50–60% of VO_2__peak_ MICT (matched-work with HIIT): 20–30 min at 60–65% of VO_2__peak_ CON: instructed to remain sedentary	4–6 (12–18)	60–90 vs. 60–90
Moholdt et al. (51)	107 (male, female)	HIIT MICT	Myocardial infarction	HIIT: 56.7 ± 10.4 MICT: 57.7 ± 9.3	HIIT: 26.8 ± 3.0 MICT: 27.2 ± 4.1	12 weeks walking/running; supervised 2 d/week; unsupervised 1 day/week	HIIT: four sets of 4-min at 85–95% of HR_max_ with 3-min recovery at 70% of HR_max_ MICT (ND matched-work with HIIT): 35-min walking, jogging, lunges and squats	16 (48)	76 vs. 120
Molmen- Hansen et al. (75)	88 (male, female)	HIIT MICT CON	Hypertensive	HIIT: 52.5 ± 7.4 MICT: 53.6 ± 6.5 CON: 51.3 ± 9.2	HIIT: 26.8 ± 4.1 MICT: 27.9 ± 3.2 CON: 28.8 ± 3.7	12 weeks uphill treadmill walking/running; supervised 3 days/week	HIIT: four sets of 4-min at 85–90% of HR_max_ with 3-min recovery at 60–70% of HR_max_ MICT (matched-work with HIIT): 47-min at 70% of HR_max_ CON: standard advice for hypertension, including regular light–moderate intensity exercise	16 (48)	114 vs. 141
Munk et al. (68)	40 (male, female)	HIIT CON	Coronary artery disease	HIIT: 57.0 ± 14.0 CON: 61.0 ± 10.0	HIIT: 27.1 ± 5.2 CON: 27.7 ± 4.5	6 months cycling; supervised 3 days/week	HIIT: four sets of 4-min at 80–90% of HR_max_ with 3-min recovery at 60–70% of HR_max_ CON: without any structured program	16 (48)	NA
Novaković et al. (76)	30 (male, female)	HIIT MICT CON	Repaired tetralogy of Fallot	HIIT: 36.2 ± 6.8 MICT: 40.1 ± 10.4 CON: 38.4 ± 8.9	HIIT: 24.5 ± 6.2 MICT: 26.3 ± 6.0 CON: 24.4 ± 5.6	36 sessions cycling; supervised 2–3 days/week	HIIT: eight sets of 1-min at 80% of HR_peak_ with 3-min recovery at 60% of HR_peak_ MICT (matched-work with HIIT): 26-min at 70% of HR_peak_ CON: regular unsupervised physical activities	8 (16–24)	84–126 vs. 82–123
Nytrøen et al. (52)	81 (male, female)	HIIT MICT	Heart transplant	HIIT: 50.0 ± 12.0 MICT: 48.0 ± 14.0	HIIT: 24.8 ± 3.4 MICT: 25.6 ± 3.9	12 months cycling; supervised 2–3 days/week	HIIT: four sets of 4-min at 85–95% of peak effort with 3-min recovery at 60–70% of peak effort MICT (matched-work with HIIT): 25-min at 60–80% of peak effort	16 (32–48)	112–168 vs. 112–168
O’Brien et al. (53)	24 (male, female)	HIIT MICT	Sedentary older adults	HIIT: 68.0 ± 5.0 MICT: 68.0 ± 6.0	HIIT: 25.9 ± 3.1 MICT: 25.2 ± 3.6	6 weeks cycling; supervised 3 days/week	SIT: 2× 35–45 sets of 15-s at 100% of peak power output with 15-s recovery MICT (matched-work with HIIT): 30–39 min at 60% of peak power output	17.5–22.5 (52.5–67.5)	135–165 vs. 120–147
Petrick et al. (54)	23 (male)	HIIT MICT	Obese and overweight	HIIT: 39.4 ± 14.9 MICT: 35.3 ± 15.1	HIIT: 34.1 ± 4.3 MICT: 33.9 ± 2.4	6 weeks cycling; supervised 3 days/week for HIIT and supervised 5 days/week for MICT	SIT: four to six sets of 30-s at 170% of maximal workload with 2-min recovery at 50 W MICT (unmatched-work with HIIT): 30–40 min at 60% of maximal workload	2–3 (6–9)	45–60 vs. 150–200
Rakobowchuk et al. (55)	20 (male, female)	HIIT MICT	Healthy	HIIT: 23.6 ± 3.2 MICT: 23.0 ± 2.4	HIIT: 23.6 ± 3.0 MICT: 24.3 ± 2.1	6 weeks cycling; supervised 3 days/week for HIIT and supervised 5 days/week for MICT	SIT: four to six sets of Wingate tests with 4.5-min recovery at 30 W MICT (unmatched-work with HIIT): 40–60 min at 65% of VO_2__peak_	2–3 (6–9)	43.5–76.5 vs. 200–300
Ramírez-Vélez et al. (69)	36 (male, female)	HIIT CON	Overweight with abdominal obesity	HIIT: 40.8 ± 7.1 CON: 40.8 ± 7.1	HIIT: 30.0 ± 3.5 CON: 30.0 ± 3.5	12 weeks treadmill walking/running; supervised 3 days/week	HIIT: four sets of 4-min at 85–95% of HR_max_ with 4-min recovery at 65% of HR_max_ CON: received nutritional advice	16 (48)	NA
Ramírez-Vélez et al. (56)	21 (ND)	HIIT MICT	Sedentary adults	HIIT: 18−45 MICT: 18−45	HIIT: 25.5 ± 4.2 MICT: 23.6 ± 3.6	12 weeks treadmill walking/running; supervised and unsupervised 3 days/week	HIIT: four sets of 4-min at 85–95% of heart rate reverse with 4-min recovery at 75–85% of heart rate reserve MICT (matched-work with HIIT): 30–35 min at 60–75% of heart rate reserve	16 (48)	114–126 vs. 132–147
Sarvasti et al. (57)	11 (male, female)	HIIT MICT	Coronary artery disease	HIIT: 48.5 ± 6.6 MICT: 48.5 ± 6.6	HIIT: 27.0 ± 3.9 MICT: 27.0 ± 3.9	2 weeks treadmill walking; supervised 3 days/week	HIIT: four sets of 4-min at 60–80% of heart rate reverse with 3-min recovery at 40–50% of heart rate reserve MICT (matched-work with HIIT): 29-min at 40–60% of heart rate reserve	16 (48)	99 vs. 111
Sawyer et al. (58)	22 (male, female)	HIIT MICT	Obese	HIIT: 35.1 ± 8.1 MICT: 35.1 ± 8.1	HIIT: 37.4 ± 6.2 MICT: 34.5 ± 3.2	8 weeks cycling; supervised 3 days/week	HIIT: ten sets of 1-min at 90–95% of HR_max_ with 1-min recovery at 25–50 W MICT (not matched-work with HIIT): 30-min at 70–75% of HR_max_	10 (30)	87 vs. 120
Schjerve et al. (59)	27 (male, female)	HIIT MICT	Obese	HIIT: 46.9 ± 8.2 MICT: 44.4 ± 7.6	HIIT: 36.6 ± 4.5 MICT: 36.7 ± 5.0	12 weeks walking/jogging; supervised 2 days/week; unsupervised 1 day/week	HIIT: four sets of 4-min at 85–95% of HR_max_ with 3-min recovery at 50–60% of HR_max_ MICT (matched-work with HIIT): 47-min at 60–70% of HR_max_	16 (48)	120 vs. 141
Stensvold et al. (70)	22 (male, female)	HIIT CON	Metabolic syndrome	HIIT: 49.9 ± 10.1 MICT: 47.3 ± 10.2	HIIT: 31.3 ± 4.3 MICT: 31.9 ± 4.1	12 weeks treadmill walking/running; supervised 3 days/week	HIIT: four sets of 4-min at 90–95% of HR_peak_ with 3-min recovery at 70% of HR_peak_ CON: instructed not to change their dietary patterns or physical activity	16 (48)	NA
Taylor et al. (60)	54 (male, female)	HIIT MICT	Coronary artery disease	HIIT: 64.0 ± 8.0 MICT: 63.0 ± 8.0	HIIT: 28.7 ± 4.3 MICT: 29.5 ± 4.2	4 weeks walking/running or cycling/elliptical; supervised 2 days/week; unsupervised 1 day/week	HIIT: four sets of 4-min at 85–95% of HR_peak_ with 3-min recovery MICT (ND matched-work with HIIT): 40-min at 65–75% of HR_peak_	16 (48)	ND
Thijssen et al. (61)	24 (male, female)	HIIT MICT	Heart failure	HIIT: 63.0 ± 8.0 MICT: 64.0 ± 8.0	HIIT: 28.1 ± 7.5 MICT: 28.9 ± 4.7	12 weeks cycling; supervised 3 days/week	HIIT: ten sets of 1-min at 90% of maximal workload by 2.5-min recovery at 30% of maximal workload MICT (matched-work with HIIT): 30-min at 60–75% of maximal workload	10 (20)	70 vs. 60
Tjønna et al. (77)	32 (male, female)	HIIT MICT CON	Metabolic syndrome	HIIT: 55.3 ± 13.2 MICT: 52.0 ± 10.6 CON: 49.6 ± 9.0	HIIT: 29.8 ± 5.5 MICT: 29.4 ± 4.9 CON: 32.1 ± 3.3	16 weeks uphill treadmill walking/running; supervised 3 days/week	HIIT: four sets of 4-min at 90% of maximal heart rate with 3-min recovery at 70% of maximal heart rate MICT (matched-work with HIIT): 47-min at 70% of maximal heart rate CON: advice from family physicians	16 (48)	120 vs. 141
Tucker et al. (78)	29 (male)	HIIT MICT CON	sedentary inactive	HIIT: 30.0 ± 7.0 MICT: 29.0 ± 7.0 CON: 28.0 ± 9.0	HIIT: 30.2 ± 3.0 MICT: 29.7 ± 4.5 CON: 29.6 ± 3.9	4 weeks cycling; supervised 4 days/week	HIIT: eight to eleven sets of 1-min at 90–95% of HR_max_ with 1-min recovery MICT (matched-work with HIIT): 30–45 min at 50% of VO_2__max_ CON: instructed to maintain their current physical activity pattern	8–11 (32–44)	100–128 vs. 160–220
Turri-Silva et al. (71)	18 (male, female)	HIIT CON	Heart failure	HIIT: 60.9 ± 9.7 CON: 56.0 ± 9.7	HIIT: 29.4 ± 5.2 CON: 28.6 ± 4.5	12 weeks cycling/running; supervised 3 days/week	HIIT: four sets of 3-min at high intensity with 4-min recovery at moderate intensity CON: maintained daily activities	12 (36)	NA
Wisløff et al. (79)	27 (male, female)	HIIT MICT CON	Heart failure	HIIT: 76.5 ± 9.0 MICT: 74.4 ± 12.0 CON: 75.5 ± 13.0	HIIT: 24.5 ± 3.0 MICT: 24.7 ± 3.0 CON: 25.5 ± 2.0	12 weeks uphill treadmill walking; supervised 2 days/week; unsupervised 1 days/week	HIIT: four sets of 4-min at 90–95% of HR_peak_ with 3-min recovery at 50–70% of HR_peak_ MICT (matched-work with HIIT): 47-min at 70–75% of HR_peak_ CON: one exercise session every 3 weeks	16 (48)	114 vs. 141

HIIT, high-intensity interval training; MICT, moderate-intensity continuous training; CON, control; VO_2__*max/peak*_, maximal or peak oxygen uptake; HR_*max/peak*_, maximal or peak heart rate; W, watt; ND, not-described; NA, not-available.

### Intervention characteristics

The intervention characteristics are described in Table 1. Briefly, most studies used cycling, and treadmill walking and running exercise modalities. The most frequent protocols for included HIIT studies comprised four sets of 4-min at 80–95% HRmax or HRR interspersed with 3- or 4-min active recovery periods of low–moderate intensity. One study used two types of HIIT (45). In addition, most included studies used HIIT, whereas SIT was used in four studies (49, 53–55) where high-intensity bouts were performed at 100% of peak power output, 170% of maximal workload, or Wingate tests were performed. Also, one study used both HIIT (2 sessions per week) and SIT (1 session per week) (63). Intervention duration ranged from two (49, 50, 57, 66) to 16 weeks (77), with 12 weeks being the most common. Frequency of training ranged from 2 to 6 sessions pre week, with 3 sessions per week being the most common. Of the 26 studies that directly compared HIIT vs. MICT (44–61, 72–79), 17 studies clearly reported that HIIT protocols were matched with MICT protocols based on energy, time, duration, and/or total work performed (45, 47, 48, 50, 52, 53, 56, 57, 59, 61, 73–79). MICT protocols included MICT at 50–75% HRmax, HRR, or VO_2__*peck/max*_ with durations ranging from 15 to 60 min. For CON groups, maintaining sedentary lifestyles, maintaining usual activities of daily living, nutrition advice, and light-intensity exercise with short durations were used.

### Meta-analysis

#### High-intensity interval training vs. moderate-intensity continuous training

**FIGURE 2:**
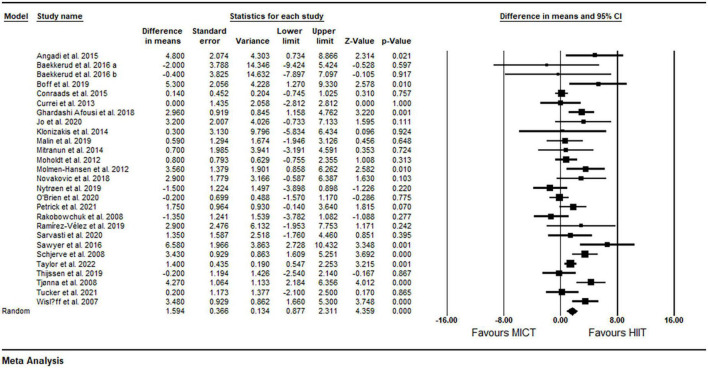
Forest plot of the effects of HIIT vs. MICT on FMD. Data are reported as WMD (95% confidence limits). FMD, brachial artery flow-mediated dilation; WMD, weighted mean differences.

Based on 27 intervention arms, HIIT effectively increased FMD [1.59% (95% CI 0.87–2.31), *p* = 0.001] when compared with MICT (Figure 2). There was significant heterogeneity amongst included studies (*I*^2^ = 61.11%, *p* = 0.001). Visual interpretation of funnel plots and Egger’s test results (*p* = 0.19) did not suggest publication bias.

##### Subgroup and sensitivity analyses

Subgroup analysis by health status revealed a significant increase in FMD in participants with metabolic disorders (WMD: 2.84%, *p* = 0.001) and cardiovascular disease (WMD: 1.24%, *p* = 0.008), but not in participants who were free of cardiometabolic diseases (WMD: −0.17%, *p* = 0.74) (Table 2). Subgroup analysis by participant BMI revealed a significant increase in FMD in participants with BMI <30 kg m^2^ (WMD: 1.24%, *p* = 0.001) and those with BMI ≥30 kg m^2^ (WMD: 2.72%, *p* = 0.001) (Table 2). Subgroup analysis based on age revealed a significant increase in FMD in participants aged <50 years (WMD: 1.65%, *p* = 0.02) and those aged ≥50 years (WMD: 1.59%, *p* = 0.001) (Table 2). Subgroup analysis based on intervention duration revealed a significant increase in FMD in both short-term (WMD: 1.36%, *p* = 0.008) and medium-term (WMD: 1.76%, *p* = 0.001) interventions (Table 2). Subgroup analysis based on the type of HIIT revealed a significant increase in FMD for LI-HIIT (WMD: 1.85 *p* = 0.001), but not for SIT (WMD: 0.16%, *p* = 0.80) (Table 2). Subgroup analysis based on volume of intense bouts of HIIT revealed a significant increase in FMD in both weekly total volume of ≤30 min (WMD: 1.58%, *p* = 0.03) and weekly total volume of >30 min (WMD: 1.62%, *p* = 0.001) (Table 2). In addition, subgroup analysis based on matching of work performed across protocols revealed a significant increase in FMD in both matched (WMD: 1.58%, *p* = 0.001) and un-matched (WMD: 1.63%, *p* = 0.008) protocols (Table 2). In addition, sensitivity analysis by omitting individual studies did not alter the significance or direction of overall results.

**TABLE 2:** Summary of subgroup analyses for the effects of HIIT vs. CON and MICT on FMD.

	Moderators	*N*	SMD (95% CI)	*P*-value	*P*-heterogeneity
**HIIT vs. CON**					
Health status	Metabolic disorders	9	3.44 (2.02–4.87)	0.001	0.001
	Cardiovascular diseases	5	4.29 (1.14–7.45)	0.008	0.001
	Free of cardiometabolic disease	3	2.73 (−0.48 to 5.96)	0.09	0.003
BMI	BMI <30	13	3.83 (2.29–5.38)	0.001	0.001
	BMI ≥30	5	3.88 (1.38–6.38)	0.002	0.001
Age	Age <50	10	3.78 (1.53–6.05)	0.001	0.001
	Age ≥50	8	3.79 (2.24–5.31)	0.001	0.001
Intervention duration	Short-term <12 week	7	3.62 (1.69–5.55)	0.001	0.001
	Medium-term ≥12 week	11	3.95 (2.18–5.72)	0.001	0.001
Total time	Time ≤30 min	5	5.69 (3.16–8.11)	0.001	0.10
	Time >30 min	13	3.24 (1.94–4.54)	0.001	0.001
**HIIT vs. MICT**					
Health status	Metabolic disorders	11	2.84 (1.77–3.90)	0.001	0.12
	Cardiovascular diseases	11	1.24 (0.32–2.17)	0.008	0.005
	Free of cardiometabolic disease	5	−0.17 (−1.19 to 0.84)	0.74	0.63
BMI	BMI <30	19	1.24 (0.48–2.00)	0.001	0.001
	BMI ≥30	7	2.72 (1.16–4.29)	0.001	0.05
Age	Age <50	12	1.65 (0.20–3.10)	0.02	0.002
	Age ≥50	15	1.56 (0.73–2.40)	0.001	0.001
Intervention duration	Short-term <12 week	14	1.36 (0.35–2.37)	0.008	0.01
	Medium-term ≥12 week	13	1.76 (0.69–2.84)	0.001	0.001
Interval types	HIIT	23	1.87 (1.08–2.66)	0.001	0.001
	SIT	4	0.16 (−1.18 to 1.51)	0.80	0.21
Total time	Time ≤30 min	11	1.58 (0.14–3.02)	0.03	0.02
	Time >30 min	16	1.62 (0.76–2.47)	0.001	0.001
Matching of work	Matched	17	1.58 (0.63–2.52)	0.001	0.001
	Un-matched	7	1.63 (0.41–2.84)	0.008	0.01

#### High-intensity interval training vs. CON

**FIGURE 3:**
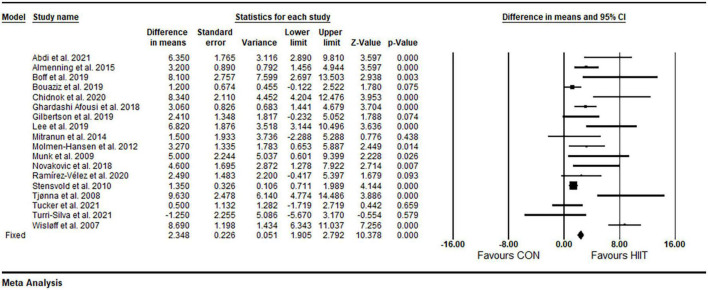
Forest plot of the effects of HIIT vs. CON on FMD. Data are reported as WMD (95% confidence limits). FMD, brachial artery flow-mediated dilation; WMD,: weighted mean differences.

Based on 18 intervention arms, HIIT effectively increased FMD [3.80% (95% CI 2.58–5.01), *p* = 0.001] when compared with CON (Figure 3). There was significant heterogeneity amongst included studies (*I*^2^ = 79.49%, *p* = 0.001). Both visual interpretation of funnel plots and Egger’s test results (*p* = 0.004) suggested publication bias. After trim and fill correction, four studies required adjustment, with overall changes of WMD and CIs being 2.73 (95% CI 1.46–3.99).

##### Subgroup and sensitivity analyses

Subgroup analysis by health status revealed a significant increase in FMD in participants with metabolic disorders (WMD: 3.44%, *p* = 0.001) and cardiovascular diseases (WMD: 4.29%, *p* = 0.008), but not in participants who were free of cardiometabolic diseases (WMD: 2.73%, *p* = 0.09) (Table 2). Subgroup analysis by participant BMIs revealed a significant increase in FMD in participants with BMIs <30 kg m^2^ (WMD: 3.88%, *p* = 0.002) and those with BMIs ≥30 kg m^2^ (WMD: 3.83%, *p* = 0.001) (Table 2). Subgroup analysis based on age revealed a significant increase in FMD in participants ages <50 years (WMD: 3.78%, *p* = 0.001) and those with age ≥50 years (WMD: 3.79%, *p* = 0.001) (Table 2). Subgroup analysis based on intervention duration revealed a significant increase in FMD in both short-term (WMD: 3.62%, *p* = 0.001) and medium-term (WMD: 3.95%, *p* = 0.001) interventions (Table 2). Subgroup analysis based on volume of intense bouts HIIT revealed a significant increase in FMD for weekly total times of ≤30 min (WMD: 5.63%, *p* = 0.001) and weekly total times of >30 min (WMD: 3.24%, *p* = 0.001) (Table 2). Because of the small number of studies for SIT, subgroup analysis was not conducted by a type of HIIT. In addition, sensitivity analysis by omitting individual studies did not alter the significance or direction of overall results.

### Quality assessment

The methodological quality of individual studies was assessed using the PEDro tool with scores ranging from 5–8 out of a maximum of 9 points. These data are summarized in Supplementary Table 2.

## Discussion

Results from current systematic review and meta-analysis show that HIIT is effective for increasing FMD by 3.80%. In addition, when compared with MICT, HIIT increased FMD by 1.59% more than MICT. From a clinical perspective, these findings have important implications for the promotion of therapeutic strategies including HIIT given the efficacy and time-efficiency of exercise training type for improving vascular function.

It is well established that exercise training is associated with beneficial cardiometabolic health effects, that are mediated by improved vascular function (80). Previous systematic reviews involving healthy participants, as well as individuals with chronic diseases, indicated that exercise training including aerobic, resistance, and combined training increase FMD (81–84), with 1% change being associated with a 13% reduction in adverse cardiovascular events (7). Our finding of 3.8% increase in FMD following HIIT is thus predicted to be clinically important, and are in accord with HIIT improving brachial artery FMD by 4.31% in patients cardiovascular and metabolic diseases (37). However, the previous meta-analysis included interventions involving HIIT vs. MICT, and the HIIT effects were assessed using paired analysis (pre- and post-intervention analysis), not compared with a control group (37). This approach (within group) did not allow the appropriate evaluation of the effects of HIIT to be elucidated, especially since most of the participants were also taking medications that are known to improve vascular function (37).

The potential mechanisms underlying the increase in FMD may be explain by greater NO bioavailability, antioxidant capacity, anti-inflammatory effects, and increased abundance of endothelial progenitor cells (84, 85). Endothelial dysfunction is characterized by decreased NO bioavailability, and HIIT enhances blood flow and shear stress, thereby increasing endothelial NO synthase activity and NO quenching, leading to improvements in NO bioavailability and endothelium-dependent vasodilation (74, 86). In addition, HIIT is associated with increases in anti-inflammatory cytokines and antioxidant enzymes, reductions in pro-inflammatory cytokines and oxidative enzymes (13, 74, 87, 88), and mobilization and functionality of endothelial progenitor cells (89), which may enable improved endothelial function.

High-intensity interval training has been considered a time-efficient mode of exercise training for several physiological adaptations such as inflammation, glycemia, fat loss and weight management, with superior or similar effects, yet less overall total exercise time (13, 17, 32, 33, 35, 37, 38, 90). The second part of the current meta-analysis investigated the effect of HIIT vs. MICT showing superior effects for HIIT on FMD by 1.59%. This is consistent with previous systematic reviews and meta-analysis showing improvements in FMD of 2.26% compared with MICT. A possible mechanism for superior effects for HIIT, relative to MICT, is a combination of direct and indirect effects of HIIT on NO bioavailability and endothelial function. HIIT showed superior effects on shear stress as compared with MICT (37, 75, 79), and shear stress is strongly correlated with NO bioavailability. In addition, greater improvements in inflammatory cytokines, antioxidants status, insulin sensitivity, and lipid profiles following HIIT relative to MICT, are among the other possible mechanisms underlying the superior effects of HIIT for increasing FMD (37). Whilst not our primary research question, we examined the effects of MICT vs. CON in the trials where HIIT, MICT, and CON were all included. Meta-analysis indicated that MICT effectively increased FMD as compared with CON [1.65% (95% CI 0.13–3.17), *p* = 03], suggesting that, despite the greater effects of HIIT, MICT exercise can also be an effective mode of training. In addition, it should be noted that comparisons between training protocols based on energy expenditure may have resulted in risk for bias given the lack of consideration of internal training load. For example, it is possible that a greater training load occurred in the HIIT protocols in comparison to MICT (91). Therefore, when interpreting the current findings, these issues should be considered.

High-intensity interval training has been considered a potent and safe intervention for achieving beneficial health outcomes by central and peripheral adaptations in healthy populations, patients with chronic cardiometabolic disorders, and those with risk for cardiometabolic disease (92–94). However, there has been uncertainty regarding the effect HIIT may have on vascular function in healthy individuals vs. clinical populations. Ramos and colleagues reported that HIIT increased FMD in patients with metabolic disorders or cardiovascular diseases (37). The current analysis extends the previous meta-analysis (37) suggesting that HIIT, relative to both MICT and CON, increases FMD in participants with both metabolic disorders and cardiovascular diseases, but not in those who were free of cardiometabolic diseases. These observations are clinically significant, as vascular dysfunction is associated with an increased risk for cardiovascular diseases, for example heart failure or coronary artery disease. In addition, vascular dysfunction is associated with increased risk for metabolic disorders such as obesity and T2D.

The current meta-analysis suggests that HIIT is effective for increasing FMD regardless of age and BMI, when compared to both MICT and CON. Aging and obesity are associated with impaired vascular endothelial function which contributes to atherosclerosis (95–97). Several mechanisms, such as reductions in NO bioavailability, increased oxidative stress, development of low-grade inflammation, and increased activity of vasoconstrictors, are involved in vascular dysfunction that occurs with aging and obesity (95–97). Taken together, our novel findings suggest that HIIT favorably increases FMD and can be considered as a strategy for mitigating vascular dysfunction. HIIT duration and volume were also considered as an important moderator that may influence HIIT- induced adaptations (13, 30, 98, 99), but it is not clear whether these factors influence improvements in vascular function. We found that HIIT interventions of both medium and short-term duration, as well as with weekly total exercise times of ≤ and >30 min, relative to MICT and CON, are effective for increasing FMD. These results indicate that it is not necessary to engage in high volumes of HIIT to derive beneficial effects for vascular function, and further, these adaptations seem to occur rapidly once engaging in HIIT training.

As with any study, the current systematic review and meta-analysis had several limitations that should be considered when interpreting the results. There was significant heterogeneity in the results. To overcome this limitation, we performed several subgroup analyses to assess the sources of heterogeneity and found that the health status of participants may be an important contributor to differential results. Finally, we were not able to examine the effects of SIT as compared to the more common types of HIIT due to the lack of studies using SIT.

## Conclusion

The current meta-analysis demonstrated that HIIT is an effective mode of exercise training for improving vascular function, particularly in those with metabolic disorders and cardiovascular diseases and is superior to MICT, suggesting HIIT is a time-efficient intervention for improving vascular function. These results hold true for low weekly volume exercise, occur relatively rapidly, and seem to be consistent across ages and BMI status.

## Perspective

High-intensity interval training is an effective mode of exercise training which can improve vascular function in adults. These benefits were observed in both older adults with metabolic disorders and cardiovascular diseases. Our results highlight that HIIT is a time-efficient for improving FMD and therefore, HIIT should be considered a viable strategy for improvement of vascular function.

## Data availability statement

The original contributions presented in this study are included in the article/Supplementary material, further inquiries can be directed to the corresponding author.

## Author contributions

MK, MHS, FK, MES, and SR conceived and designed the study. MK, MHS, and FK carried out the screenings and reviews. MK and MHS analyzed the manuscript. MK drafted the manuscript. MES and SR revised the manuscript. All authors read and approved the final manuscript.
