# Supplementary material for: The impact of high-intensity interval training on vascular function in adults: A systematic review and meta-analysis
Source: Front Cardiovasc Med. 2022 Nov 17;9:1046560. doi: 10.3389/fcvm.2022.1046560 (PMC9713318; doi:10.3389/fcvm.2022.1046560)
Supplement: Supplementary file 1 [file Data_Sheet_1.docx]

**Supplementary Table 1.** Search strategy

| Databases | Search strategy | Limits | Results |
| --- | --- | --- | --- |
| PubMed | ("high intensity interval training"[All Fields] OR "high intensity interval exercise"[All Fields] OR "high intensity intermittent training"[All Fields] OR "high intensity intermittent exercise"[All Fields] OR "aerobic interval training"[All Fields] OR "aerobic interval exercise"[All Fields] OR "interval training"[All Fields] OR "interval exercise"[All Fields] OR "sprint interval training"[All Fields] OR "sprint interval exercise"[All Fields]) AND ("brachial artery"[All Fields] OR "brachial artery dilation"[All Fields] OR "flow mediated dilation"[All Fields] OR "endothelial function"[All Fields] OR "endothelium"[All Fields] OR "artery blood flow"[All Fields] OR "artery dilatation"[All Fields] OR "flow-mediated"[All Fields] OR "flow-mediated"[All Fields] OR "vascular"[All Fields] OR "vascular endothelium"[All Fields] OR "vascular endothelium"[All Fields] OR "vascular reactivity"[All Fields] OR "vasodilation"[All Fields]) | Humans, English | 314 |
| Scopus | ( TITLE-ABS-KEY ( "high intensity interval training" OR "high intensity interval exercise" OR "high intensity intermittent training" OR "high intensity intermittent exercise" OR "aerobic interval training" OR "aerobic interval exercise" OR "interval training" OR "interval exercise" OR "sprint interval training" OR "sprint interval exercise" ) AND TITLE-ABS-KEY ( "brachial artery" OR "brachial artery dilation" OR "flow mediated dilation" OR "endothelial function" OR "endothelium" OR "artery blood flow" OR "artery dilatation" OR "flow-mediated" OR "flow mediated" OR " vascular" OR "vascular endothelium" OR "vascular endothelium" OR "vascular reactivity" OR "vasodilation" ) | Article, English | 454 |
| Web of science | (ALL=("high intensity interval training" or "high intensity interval exercise" or "high intensity intermittent training" or "high intensity intermittent exercise" or "aerobic interval training" or "aerobic interval exercise" or "interval training" or "interval exercise" or "sprint interval training" or "sprint interval exercise")) AND ALL=(“brachial artery” or “brachial artery dilation” or “flow mediated dilation” or “endothelial function” or “endothelium” or “artery blood flow” or “artery dilatation” or “flow-mediated” or “flow mediated” or “ vascular” or “vascular endothelium” or “vascular endothelium” or “vascular reactivity” or “vasodilation”) | Article, English | 386 |

**Supplementary Table 2.** Risk of bias assessment

| **Authors & Year** | **Criteria 1** | **Criteria 2** | **Criteria 3** | **Criteria 4** | **Criteria 5** | **Criteria 6** | **Criteria 7** | **Criteria 8** | **Criteria 9** | **total** |
| --- | --- | --- | --- | --- | --- | --- | --- | --- | --- | --- |
| Abdi et al. 2021[62] | ✓ | ✓ | 🗶 | ✓ | 🗶 | ✓ | 🗶 | ✓ | ✓ | 6 |
| Almenning et al. 2015[63] | ✓ | ✓ | ? | ✓ | 🗶 | ✓ | 🗶 | ✓ | ✓ | 6 |
| Angadi et al. 2015[44] | ✓ | ✓ | 🗶 | ✓ | ✓ | 🗶 | 🗶 | ✓ | ✓ | 6 |
| Baekkerud et al. 2016[45] | ✓ | ✓ | ? | ✓ | 🗶 | ✓ | 🗶 | ✓ | ✓ | 6 |
| Boff et al. 2019[72] | ✓ | ✓ | ✓ | ✓ | ✓ | 🗶 | 🗶 | ✓ | ✓ | 7 |
| Bouaziz et al. 2019[64] | ✓ | ✓ | ✓ | ✓ | ✓ | ✓ | 🗶 | ✓ | ✓ | 8 |
| Chidnok et al. 2020[65] | ✓ | ✓ | 🗶 | ✓ | 🗶 | ✓ | 🗶 | ✓ | ✓ | 6 |
| Currie et al. 2013[46] | ✓ | ✓ | 🗶 | ✓ | 🗶 | ✓ | 🗶 | ✓ | ✓ | 6 |
| Conraads et al. 2015[47] | ✓ | ✓ | 🗶 | ✓ | ✓ | 🗶 | ✓ | ✓ | ✓ | 7 |
| Ghardashi Afousi et al. 2018[73] | ✓ | ✓ | 🗶 | ✓ | ✓ | 🗶 | 🗶 | ✓ | ✓ | 6 |
| Gilbertson et al. 2019[66] | ✓ | ✓ | 🗶 | ✓ | ✓ | ✓ | 🗶 | ✓ | ✓ | 7 |
| Jo et al. 2020[48] | ✓ | ✓ | 🗶 | ✓ | ✓ | ✓ | 🗶 | ✓ | ✓ | 7 |
| Klonizakis et al. 2014[49] | ✓ | ✓ | ✓ | ✓ | 🗶 | ✓ | 🗶 | ✓ | ✓ | 8 |
| Lee et al. 2019[67] | ✓ | ✓ | 🗶 | ✓ | 🗶 | ✓ | 🗶 | ✓ | ✓ | 6 |
| Malin et al. 2019[50] | ✓ | ✓ | 🗶 | ✓ | ✓ | ✓ | 🗶 | ✓ | ✓ | 7 |
| Mitranun et al. 2014[74] | ✓ | ✓ | 🗶 | ✓ | 🗶 | ✓ | 🗶 | ✓ | ✓ | 6 |
| Moholdt et al. 2012[51] | ✓ | ✓ | 🗶 | ✓ | ✓ | 🗶 | ✓ | ✓ | ✓ | 7 |
| Molmen-Hansen et al. 2012[75] | ✓ | ✓ | 🗶 | ✓ | ✓ | 🗶 | 🗶 | ✓ | ✓ | 6 |
| Munk et al. 2009[68] | ✓ | ✓ | 🗶 | ✓ | ✓ | ✓ | 🗶 | ✓ | ✓ | 7 |
| Novaković et al. 2018[76] | ✓ | ✓ | ✓ | ✓ | 🗶 | ✓ | 🗶 | ✓ | ✓ | 7 |
| Nytrøen et al. 2019[52] | ✓ | ✓ | 🗶 | ✓ | ✓ | ✓ | 🗶 | ✓ | ✓ | 7 |
| O'Brien et al. 2020[53] | 🗶 | ✓ | 🗶 | ✓ | ✓ | ✓ | 🗶 | ✓ | ✓ | 6 |
| Petrick et al. 2021[54] | ✓ | ✓ | 🗶 | ✓ | 🗶 | ✓ | 🗶 | ✓ | ✓ | 6 |
| Rakobowchuk et al. 2008[55] | ✓ | ✓ | 🗶 | ✓ | 🗶 | ✓ | 🗶 | ✓ | ✓ | 6 |
| Ramírez-Vélez et al. 2020[69] | ? | ✓ | ✓ | ✓ | 🗶 | 🗶 | 🗶 | ✓ | ✓ | 5 |
| Ramírez-Vélez et al. 2019[56] | ✓ | ✓ | ✓ | ✓ | ✓ | ✓ | 🗶 | ✓ | ✓ | 8 |
| Sarvasti et al. 2020[57] | ✓ | ✓ | 🗶 | ✓ | 🗶 | ✓ | 🗶 | ✓ | ✓ | 6 |
| Sawyer et al. 2016[58] | ✓ | ✓ | 🗶 | ✓ | ✓ | ✓ | 🗶 | ✓ | ✓ | 7 |
| Schjerve et al. 2008[59] | ✓ | ✓ | 🗶 | ✓ | ✓ | 🗶 | 🗶 | ✓ | ✓ | 6 |
| Stensvold et al. 2010[70] | ✓ | ✓ | ✓ | ✓ | 🗶 | ✓ | 🗶 | ✓ | ✓ | 7 |
| Taylor et al. 2022[60] | ✓ | ✓ | 🗶 | ✓ | ✓ | ✓ | ✓ | ✓ | ✓ | 8 |
| Thijssen et al. 2019[61] | ✓ | ✓ | 🗶 | ✓ | ✓ | ✓ | 🗶 | ✓ | ✓ | 7 |
| Tjønna et al. 2008[77] | ✓ | ✓ | 🗶 | ✓ | 🗶 | ✓ | 🗶 | ✓ | ✓ | 6 |
| Tucker et al. 2021[78] | ✓ | ✓ | 🗶 | ✓ | ✓ | ✓ | 🗶 | ✓ | ✓ | 7 |
| Turri-Silva et al. 2021[71] | ✓ | ✓ | ✓ | ✓ | ? | ✓ | 🗶 | ✓ | ✓ | 7 |
| Wisløff et al. 2007[79] | ✓ | ✓ | 🗶 | ✓ | 🗶 | ✓ | 🗶 | ✓ | ✓ | 6 |

(1) Eligibility Criteria specified, (2) Random allocation of participants, (3) Allocation concealed, (4) Groups similar at baseline, (5) Assessors blinded, (6) Outcome measures assessed in 85% of participants, (7) Intention to treat analysis, (8) Reporting of between group statistical comparison, (9) Point measures and measures of variability reported for main effects. ‘low (✓), ‘high (x) and unclear (?)
